# Prevention of Intestinal Allergy in Mice by rflaA:Ova Is Associated with Enforced Antigen Processing and TLR5-Dependent IL-10 Secretion by mDC

**DOI:** 10.1371/journal.pone.0087822

**Published:** 2014-02-07

**Authors:** Stefan Schülke, Sonja Wolfheimer, Gabriele Gadermaier, Andrea Wangorsch, Susanne Siebeneicher, Peter Briza, Ingo Spreitzer, Dirk Schiller, Bettina Loeschner, Satoshi Uematsu, Bernard Ryffel, Shizuo Akira, Zoe Waibler, Stefan Vieths, Masako Toda, Stephan Scheurer

**Affiliations:** 1 Division of Allergology, Paul-Ehrlich-Institut, Langen, Hessen, Germany; 2 Christian Doppler Laboratory for Allergy Diagnosis and Therapy, Department of Molecular Biology, University of Salzburg, Salzburg, Austria; 3 Junior Research Group “Experimental Allergy Models”, Paul-Ehrlich-Institut, Langen, Hessen, Germany; 4 Division of Microbiology, Paul-Ehrlich-Institut, Langen, Hessen, Germany; 5 Department of Host Defense, Research Institute for Microbial Diseases, Osaka University, Osaka, Osaka Prefecture, Japan; 6 CNRS Department of Molecular and Experimental Immunology and Neurogenetics, Allergy and Lung Inflammation, University of Orléans, Orléans, Loiret département, France; 7 Junior Research Group “Novel vaccination strategies and early immune responses”, Paul-Ehrlich-Institut, Langen, Hessen, Germany; National Council of Sciences (CONICET), Argentina

## Abstract

Conjugated vaccines consisting of flagellin and antigen activate TLR5 and induce strong innate and adaptive immune responses. Objective of the present study was to gain further insight into the mechanisms by which flagellin fusion proteins mediate their immune modulating effects. In a mouse model of Ova-induced intestinal allergy a fusion protein of flagellin and Ova (rflaA:Ova) was used for intranasal and intraperitoneal vaccination. Aggregation status of flaA, Ova and flaA:Ova were compared by light scattering, uptake of fluorescence labeled proteins into mDC was analyzed, processing was investigated by microsomal digestion experiments. Mechanism of DC-activation was investigated using proteasome and inflammasome inhibitors. Immune responses of wildtype, IL-10^−/−^, TLR5^−/−^ mDCs and Ova-transgenic T cells were investigated. Mucosal and i.p.-application of rflaA:Ova were able to prevent allergic sensitization, suppress disease-related symptoms, prevent body weight loss and reduction in food uptake. Intranasal vaccination resulted in strongest suppression of Ova-specific IgE production. These protective effects were associated with increased aggregation of rflaA:Ova and accompanied by tenfold higher uptake rates into mDC compared to the mixture of both proteins. Microsomal digestion showed that stimulation with rflaA:Ova resulted in faster degradation and the generation of different peptides compared to rOva. rflaA:Ova-mediated activation of mDC could be suppressed in a dose-dependent manner by the application of both inflammasome and proteasome inhibitors. Using TLR5^−/−^ mDC the rflaA:Ova induced IL-10 secretion was shown to be TLR5 dependent. In co-cultures of IL-10^−/−^ mDC with DO11.10 T cells the lack of rflaA:Ova-mediated IL-10 secretion resulted in enhanced levels of both TH2 (IL-4, IL-5) and TH1 (IL-2 and IFN-y) cytokines. In summary, mucosal vaccination with flaA:Ova showed strongest preventive effect. Stimulation with rflaA:Ova results in strong immune modulation mediated by enhanced uptake of the aggregated fusion protein, likely resulting in a different processing by DC as well as stronger TLR5 mediated cell activation.

## Introduction

Bacterial flagellins, including flaA derived from *L. monocytogenes*, were identified as the ligands for TLR5 [1], [2], shown to be pro-inflammatory at the picomolar range and have strong immune modulatory activities [3]–[7]. Despite its undoubted immune stimulatory potential, the actual profile of flagellin induced T helper cell responses remains a matter of controversial discussion. Previous reports using flagellin as an adjuvant showed either inhibition of TH2 responses without inducing a TH1 response [7] or induction of polarized TH2 responses by suppressing TH1 responses [5]. For example treatment of BALB/c mice, intranasally sensitized to the chicken egg white allergen Ova, with a mixture of *V. vulnificus* flagellin B and Ova significantly inhibited subsequent Ova-induced airway hyperreactivity, airway inflammation, and TH2-cytokine production [7]. In contrast, *in vivo Salmonella* flagellin C application stimulated strong TLR5 dependent allergic airway responses to inhaled Ova and primed allergic responses to natural indoor allergens present in house dust extracts [8].

However, antigens conjugated with flagellin were shown to be effective vaccines promoting antigen-specific TH1 responses *in vivo* [9]–[14]. Conjugated flagellin vaccines have several advantages over the mixture of antigen with flagellin: Covalent fusion of flagellin and antigen is likely to result in targeted co-delivery of both antigen and adjuvant to the same APC in the context of the flagellin-mediated cell activation. On the contrary, the mixture of flagellin and antigen may be taken up independently by different cells. Furthermore, even if a single cell internalizes both proteins, the flagellin:antigen ratios and therefore the type of immune response induced will be different from cell to cell. In a worst case scenario this may result in bystander activation of antigen-specific TH2-responses by flagellin activated cells, as observed for the flagellin induced priming of allergic responses to house dust extracts [8].

In line with these considerations, flagellin antigen fusion proteins were shown to significantly increase immunogenicity and protective capacity against the target antigen [12], [15]–[18]. In previous studies we showed that fusion of *L. monocytogenes* flagellin A (flaA) and the model allergen Ova resulted in strongly enhanced mDC activation, mDC derived cytokine secretion, a suppression of TH1 and TH2 cytokine secretion from Ova-specific CD4 T cells and prevention of an allergic phenotype upon intraperitoneal application [9]. Remarkably, the co-administration of both components as a mixture did not result in similar effects. Concordantly, in an independent study Bates *et al.* reported a (*Salmonella*) flagellinC:Ova fusion protein to have a superior ability to induce IL-2 secretion, T cell clustering, and T cell proliferation compared to the equimolar mixture of both components [10]. Here, enhanced T cell activation was suggested to be mediated by a combination of antigen-targeting to TLR5 expressing DC and flagellin-induced signaling via TLR5 and MyD88 [10]. However, the exact mechanism of flagellin fusion protein mediated DC activation, likewise the internalization and processing by which the flagellin component exerts its adjuvant activity, remain to be clarified.

In the present study we showed that intranasal application of rflaA:Ova fusion protein targeting theTLR5-expressing cells of the mucosa was even more efficient in suppressing Ova-specific IgE production and preventing Ova-induced intestinal allergic reactions than intraperitoneal application. Furthermore, we investigated whether structural features of such modular vaccines might affect antigen internalization, processing, the induction and type of antigen specific innate and adaptive immune responses.

## Methods

### Generation and Characterization of Recombinant Flagellin, rOva, and rflaA:Ova Fusion Protein

Generation and purification, endotoxin depletion, and TLR5-activation capacity of rflaA (X65624), rOva (NM205152), and rflaA:Ova fusion protein were performed as previously described [19].

### Dynamic Light Scattering Analysis

Dynamic light scattering analysis was performed using a Zetasizer Nano ZS (Malvern, Herrenberg, Germany). For light scattering analysis 70 µl of rflaA (300 µg/ml), rflaA:Ova (690 µg/ml) and rOva (1 mg/ml) in PBS were analyzed at room temperature. Three individual measurements per protein were performed and mean frequencies (as numbers [relative % in class]) of hydrodynamic radii (R_H_) in nm were plotted.

### Mice

BALB/c and Ova-TCR transgenic DO11.10 mice (BALB/c background, Jackson Laboratories, Bar Harbor, Maine, USA), C57BL/6 mice (Harlan, Rossdorf, Germany), IL-10^−/−^ mice (BALB/c background), Ova-TCR transgenic OT-II and TLR5^−/−^, MyD88^−/−^, Trif^−/−^, MyD88^−/−^Trif^−/−^ mice (all C57BL/6 background) were bred at the animal facility of the Paul-Ehrlich-Institut and kept under specified pathogen free conditions.

### Ethics Statement

Animal work was approved by the local authority (Regierungspräsidium Darmstadt, Permit Number: F107/81) and conducted in compliance with regulations of German animal welfare. All efforts were made to minimize suffering.

### Prophylactic Vaccination in a Mouse Model of Intestinal Allergy

For prophylactic vaccination BALB/c mice (female, 8–12 weeks, n = 6 per group) were treated twice in a one week interval by i.p.-injection with equimolar amounts of Ova (10 µg, Grade V, Sigma, Steinheim, Germany) or rflaA:Ova (17 µg) in a total volume of 200 µl sterile PBS, or PBS alone as control. For intranasal application of the fusion protein 17 µg of rflaA:Ova were applied 3 times every 3 days intranasally in a volume of 30 µl.

**Figure 1:**
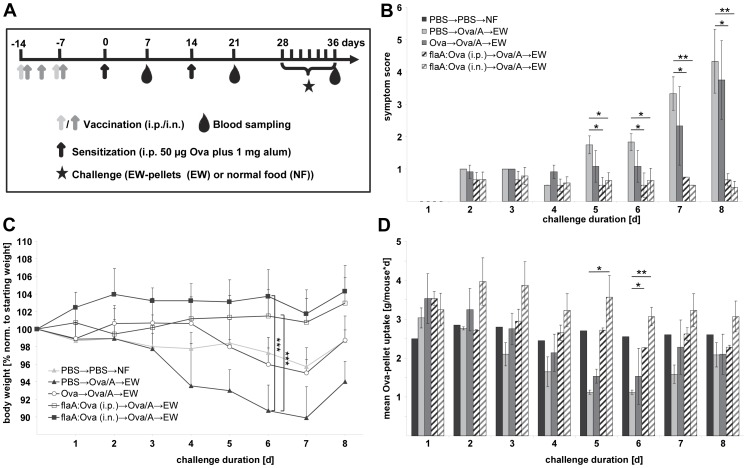
Intranasal and intraperitoneal vaccination with rflaA:Ova prevent Ova-induced intestinal allergy. Control (PBS→PBS→NF), Ova-sensitized but unvaccinated animals (PBS→Ova/A→EW), Ova-vaccinated (Ova→Ova/A→EW) and rflaA:Ova-vaccinated (flaA:Ova→Ova/A→EW) animals were continuously challenged with Ova-containing pellets (EW) for 8 days (**A**). Mean symptom scores (**B**), body weight normalized to the individual body weight before challenge (**C**), and mean EW-pellet uptake per mouse and day (**D**), were analyzed (n = 6 mice per group). NF: normal food.

One week after the last vaccination, mice were sensitized to Ova twice in a bi-weekly interval by i.p.-injection of 50 µg Ova (Grade V) absorbed to 1 mg aluminum-hydroxide adjuvant (Pierce, Solingen, Germany) in 200 µl sterile PBS (Ova/A). For induction of intestinal allergy, two weeks after the second sensitization mice were challenged by feeding egg white (EW) diet containing Ova for 8 days or feeding a conventional diet (CN) free from Ova as control (Group nomenclature: vaccination→sensitization→challenge) [20]. Blood samples were collected 1 week after each sensitization by punctuating the tail vein and on the final day of EW diet by cardiac puncture under deep ketamin/rompun anaesthesia (Fig. 1A).

### Evaluation of Clinical Symptoms

For the determination of symptom scores, individual mice were assessed for softness of faeces (scores: 0 normal; 1 soft; 2 fluid; 3 mucus-like) and phenotype (scores: 0 normal fur; 1 slightly ruffed fur; 2 strongly ruffed fur) on a daily basis (see [9]). Overall symptom scores were calculated in the range from 0 to 5. Mean symptom scores were calculated for each day and group. Exemplary data of faeces conditions and phenotypes used to generate the symptom score are depicted in Fig. S1. Moreover, body weight, and food uptake were monitored daily.

### Detection of Ova-specific IgG1, IgG2a and IgE Levels in Mouse Sera

For determination of Ova-specific IgG1, IgG2a, and IgE antibody titers in mouse sera, ELISA plates (Greiner Bio-One, Solingen-Wald, Germany) were coated with 5 µg/well Ova (Ova Grade V, Sigma,) in coating buffer (50 mM NaCO3, pH 9.6) over night at 4°C. Serum samples (50 µl each) were diluted by serial dilution (for IgE: 1×1∶10, then 6×1∶5, for IgG1 and IgG2a: 1×1∶100, then 6×1∶10) and incubated at 4°C overnight (IgE) or for 2 h at room temperature (IgG1, IgG2a). Levels of Ova-specific antibodies were measured using 50 µl secondary detection antibody diluted in PBS supplemented with 10% FCS (IgE: rat anti-mouse IgE biotin-conjugated, BD Biosciences, Heidelberg, Germany, IgG1: goat anti-mouse IgG1γ1 HRP-conjugated, 1∶8000, IgG2a: rabbit anti-mouse IgG2a, 1∶8000, both Invitrogen, Darmstadt, Germany, incubation time: 1.5 h for IgG1 and IgG2a, overnight for IgE) in combination with a streptavidin-HRP antibody (for IgE detection, 50 µl diluted 1∶2000 in PBS supplemented with 10% FCS, BD Biosciences) applied for 30 minutes at room temperature. Visualization was performed with 100 µl/well TMB substrate solution (BD Biosciences) for up to 30 minutes. The reaction was stopped by addition of 50 µl/well 25% sulfuric acid and analyzed using a SpectraMAX340PC (Molecular Devices, California, USA) reading the absorbance at 450 nm. Data were analyzed using Excel and SigmaPlot V11.0 (Systat Software, Erkrath, Germany).

### *In vitro* Generation of Murine Bone Marrow-derived Dendritic Cells

Murine mDC were generated as described previously [19]. Briefly, bone marrow cells (BMCs) were isolated from femur and tibia of BALB/c, C57BL/6, IL-10^−/−^, MyD88^−/−^, Trif^−/−^, MyD88^−/−^Trif^−/−^ and TLR5^−/−^ mice and differentiated into myeloid dendritic cells (mDC) using GM-CSF (R&D Systems, Minneapolis, USA). On day 8 mDC were used for co-culture experiments.

### Preparation of CD4^+^ T Cells and DC:T Cell Co-cultures

Splenic CD4^+^ T cells were isolated from Ova-TCR transgenic (DO11.10 and OT-II) mice using the CD4 T Cell Isolation Kit (Miltenyi Biotec, Bergisch Gladbach, Germany). Knock out (IL-10^−/−^, TLR5^−/−^, MyD88^−/−^, Trif^−/−^, or MyD88^−/−^Trif^−/−^) and respective wild type mDC (3.2×10^5^ cells/ml) were co-cultured either alone or with CD4^+^ T cells (8.0×10^5^ cells/ml, >95% purity) and stimulated with rflaA:Ova (1.7 and 17 µg/ml) for 72 h. Subsequently, concentrations of IL-2 (after 24 h), and IL-4, IL-5, IL-6, IL-10, and IFN-γ (after 72 h) in the supernatants were measured by ELISA.

### Antigen Uptake Assays

Recombinant proteins were labeled using the Alexa Flour 488 Microscale Protein Labeling Kit (Invitrogen) according to the manufacturers recommendations. Comparable degrees of fluorescence staining were adjusted by determining the protein concentration by absorbance at 280 nm and 494 nm using a Nanodrop ND-1000 (Nano-Drop Technologies, Rockland, Delaware, USA) according to the manufacturers recommendations and calculating the degree of labeling with the following formula: DOL = (A_494_×dilution factor)/(71000× protein conc. [M]). Protein concentrations after Alexa Flour 488 labeling were confirmed using BCA (Micro BCA Protein Assay Kit, Pierce, Rockford, Illinois, USA). Subsequently, 5×10^5^ BALB/c mDC/ml were stimulated with equimolar amounts of the labeled proteins (rOva, rflaA, rflaA+rOva and rflaA:Ova) for 15 minutes at 37°C, extensively washed with FACS buffer (PBS, 1% BSA, 0.3% sodium azide, 24 mM EDTA, pH 8.0). Unspecific binding was blocked by incubation of the cells with FC-Block (eBiosciences, Frankfurt, Germany) for 30 minutes, and then analyzed for protein uptake in CD11c^+^CD11b^+^B220^+^ mDC by FACS.

### Microsomal Digestion

For simulation of intracellular processing equimolar amounts of rOva and rflaA:Ova were incubated with microsomes isolated from bone marrow derived BALB/c mDC for 1 to 48 h [21], [22]. Protein digestion was monitored by SDS-PAGE (equimolar to 2.5 µg rOva per time point and lane) and Coomassie staining. Peptides derived from microsomal digestion were analyzed by mass spectrometry [21], [22] and obtained peptide sequences plotted against the respective full length sequences.

### Inhibition of Inflammasome and Proteasome Activation

To investigate the contribution of inflammasome and proteasome activation to the rflaA:Ova mediated mDC activation BALB/c mDC (3.2×10^5^ cells/ml) were preincubated for 2 h with the different inhibitors and subsequently stimulated with either rflaA:Ova (4 µg/ml) or LPS (10 µg/ml) for additional 22 h. For inhibition of inflammasome activation Z-VAD and glyburide were used, for inhibition of proteasomal degradation lactacystin was applied according to the manufacturers recommendations (all Sigma). Endosomal acidification was inhibited by addition of chloroquine (Sigma).

### Monocyte Activation Test

Monocyte activation tests (MAT) were performed as described [23], [24]. Briefly, 5 µl of freshly drawn blood from healthy volunteers were stimulated with the indicated equimolar protein amounts for 24 h in a total volume of 260 µl RPMI.

### Cytokine ELISAs

The early T cell cytokine IL-2 was determined after 24 h of stimulation, whereas IL-4 and IFN-y, secreted later after T cell stimulation as well as IL-6 and IL-10, were determined 72 h post stimulation. All murine cytokines were quantified using the BD OptEIA™ ELISA Sets (BD Biosciences). Cytokine levels (IL-1ß and IL-6) in supernatants from human MAT were determined using R&D Duo sets (R&D Systems, Wiesbaden, Germany).

### Statistical Analysis

Comparison between different treatment groups was performed by means of a mixed linear model with fixed factor *treatment group* and random factor *assay* (up to 3 assays with each two replicates). Confidence intervals for the estimated differences between treatment groups as well as p-values were either adjusted using the Wilcoxon signed-rank test (only for comparison of symptom scores), t-test (for comparison of antibody titers) or the Bonferroni method (all other tests) in order to restrict the overall type I error α (false positive results, i.e., false significant differences) to 5%. P-values <0.05, <0.01, and <0.001 were designated with *, **, and *** respectively. The statistical analyses were performed with SAS®/STAT software, version 9.2, SAS System for Windows.

## Results

### Intranasal Application of rflaA:Ova Prevents Allergic Sensitization in a Murine Model of Ova-induced Intestinal Allergy

The efficacy of intranasal flaA:Ova application to prevent allergic sensitization was compared to intraperitoneal administration using a mouse model of Ova-induced intestinal allergy (Fig. 1A, [20]). Both, intraperitoneal and intranasal vaccination with the fusion protein, but not i.p. vaccination with Ova alone, were able to prevent allergic sensitization characterized by significantly reduced symptom scores (Fig. 1B), prevention of body weight loss (Fig. 1C) and unchanged food uptake (Fig. 1D). On day 7 of EW-pellet challenge, which refers to the acute phase of the allergic response, symptom scores for rflaA:Ova vaccinated animals were 4.4 times lower (i.p. vaccination, mean score: 0.75) and 6.7 fold (i.n. vaccination, mean score: 0.5), respectively, than for the unvaccinated control group (Fig. 1B, mean score: 3.33). In contrast, i.p. vaccination with Ova alone did not result in a significant reduction of disease symptoms (Fig. 1B, mean score: 2.33). In line with this, severe intestinal symptoms determined as either fluid or mucus-like faeces (see Fig. S1: faeces scores 2 and 3 respectively) in combination with body weight loss and reduced food uptake were only observed in unvaccinated or rOva vaccinated groups but not in rflaA:Ova vaccinated animals (either i.n. or i.p.).

**Figure 2:**
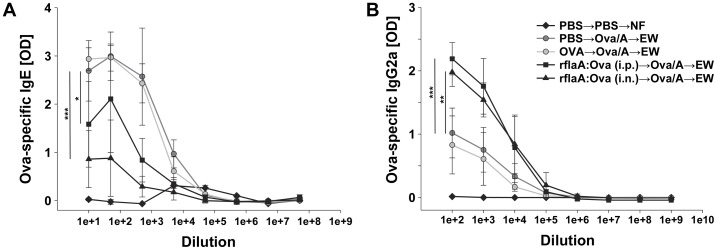
Prophylactic vaccination with rflaA:Ova induces IgG2a and suppresses IgE production. Sera of control (PBS→PBS→NF) and Ova-sensitized, EW-challenged mice (Ova/A→EW) were collected at the end of EW-pellet feeding (day 36) and analyzed for Ova-specific IgE (**A**) and Ova-specific IgG2a (**B**) antibody levels by ELISA (n = 6 mice per group).

Moreover, body weight loss observed in the unvaccinated allergic control group (Fig. 1C, 89.9% of starting weight) was prevented in mice vaccinated with the fusion protein either i.p. (Fig. 1C, 100.75% of starting weight) or i.n. (Fig. 1C, 101.75% of starting weight). Body weight loss in allergic and Ova-vaccinated mice was reflected by a reduction in Ova pellet uptake, which was not observed in mice vaccinated with the fusion protein (i.n. or i.p., Fig. 1D). When analyzing the humoral immune response of the animals, vaccination with the fusion protein applied both i.p. or i.n. resulted in a reduced production of Ova-specific IgE antibodies (Fig. 2A, Fig. S2). Here, i.n. application of the fusion protein proved to be superior in suppressing IgE production to the previously evaluated i.p. route (Fig. 2A, Fig. S2, [9]). Moreover, i.p. as well as i.n. vaccination with the fusion protein led to a strongly enhanced Ova-specific IgG2a antibody production which was not observed in the respective control groups (Fig. 2B, Fig. S2). Here, no difference between the two application routes was observed.

### rflaA:Ova Forms High Molecular Aggregates which Result in Stronger Uptake as well as Different Processing in mDC Derived Microsomes

**Figure 3:**
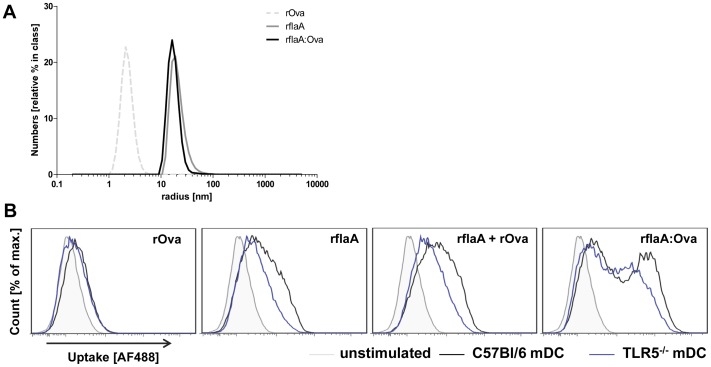
rflaA:Ova forms aggregates which are efficiently internalized by mDC. Hydrodynamic radii of rOva, rflaA and rflaA:Ova were determined via lightscattering (**A**). Uptake of Alexa Flour 488 labelled proteins into C57Bl/6 wt (black) and TLR5^−/−^ (blue) CD11c^+^CD11b^+^B220^-^ mDC stimulated with rOva (1 µg/ml), rflaA (0.7 µg/ml), rflaA (0.7 µg/ml)+rOva (1 µg/ml) or rflaA:Ova (1.7 µg/ml) was quantified 15 min post stimulation via flow cytometry (**B**).

To further investigate the mechanism by which flagellin containing fusion proteins mediate their strong immune modulating properties aggregation, internalization and antigen processing were analyzed for the flaA-conjugated fusion protein, flaA, Ova and the mixture of both components (Fig. 3). Light scattering analysis was applied to determine the hydrodynamic radii (R_H_) of the different proteins (Fig. 3A). In line with our observation that both rflaA and rflaA:Ova tend to form aggregates (unpublished observation) the radii determined for rflaA and rflaA:Ova were 9.05 fold (rflaA) and 7.82 fold (rflaA:Ova) higher than for rOva (determined mean R_H_: rOva: 2.09 nm, rflaA: 18.92 nm, rflaA:Ova: 16.34 nm, Fig. 3A).

To investigate whether these differences in molecule size had an impact on protein internalization we determined the uptake of equimolar amounts of Alexa Flour 488 labeled proteins into C57Bl/6 wild type and TLR5^−/−^ mDC (Fig. 3B). All proteins were readily taken up with overall uptake levels being slightly higher for rflaA than for rOva (Fig. 3B). In this experimental setting the observed uptake level for rflaA plus rOva in wt mDC corresponded to the cumulative signal observed for both protein administered individually (Fig. 3B). In contrast, stimulation with the equimolar amount of rflaA:Ova resulted in an approximately 10 fold higher protein uptake compared to the equimolar mixture of both proteins (Fig. 3B). In comparison to wt mDC the uptake level of both rflaA and the rflaA:Ova were reduced but not completely abrogated in TLR5-deficient mDC. As expected rOva uptake showed no difference between wild type and TLR5-deficient cells (Fig. 3B).

**Figure 4:**
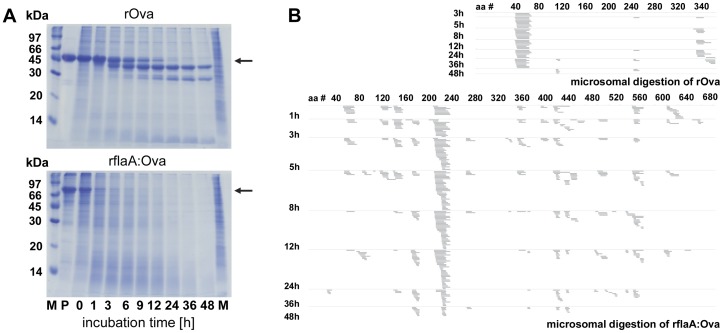
rflaA:Ova is differently processed in mDC-derived microsomes. Microsomes were isolated from unstimulated BALB/c mDC and used for digestion of rOva and rflaA:Ova (**A+B**). Protein amounts equimolar to 5 µg rOva (5 µg rOva, 8.5 µg rflaA:Ova) were digested for each indicated time point. Subsequently, half of the samples were analyzed by SDS-PAGE (**A**) and for the resulting peptide pattern by mass spectrometry B). M = molecular weight marker, arrow indicates the MW of the digested target protein. M molecular weight marker, P protein without microsomes, M microsomes without protein, aa# amino acid residue number.

Furthermore, microsomal digestion of rOva and rflaA:Ova was applied to simulate intraendosomal degradation and compare subsequent generation of Ova-derived peptides between the two proteins (Fig. 4). Analysis of the degradation products via SDS-PAGE showed that fusion of flaA to Ova resulted in faster degradation compared to the same protein amount of rOva alone (Fig. 4A). Whereas for rOva distinct degradation products (apparent molecular mass less than 45 kDa) could be detected after 48 h of microsomal digestion (Fig. 4A), microsomal digestion of rflaA:Ova resulted in almost no detectable native protein after 3 to 6 h of microsomal treatment (Fig. 4A). Analysis of the resulting peptides via mass spectrometry revealed two major peptides generated from rOva (amino acids 40 to 60 and 340 to 380, the later one corresponding to the described OT-I epitope ISQAVHAAHAEINEAGR, Fig. 4B). In contrast, microsomal digestion of rflaA:Ova resulted in Ova-derived peptides that were scattered over the whole length of the Ova part of the fusion protein. No distinct clusters were observed for rOva derived peptides (Fig. 4B). Remarkably, processing of flagellin showed very prominent clusters of peptides (aa residues 209 to 236) which could be detected as early as 1 h after starting the digestion (Fig. 4B).

### rflaA:Ova Mediated Cell Activation Depends on the Inflammasome and the Proteasome

**Figure 5:**
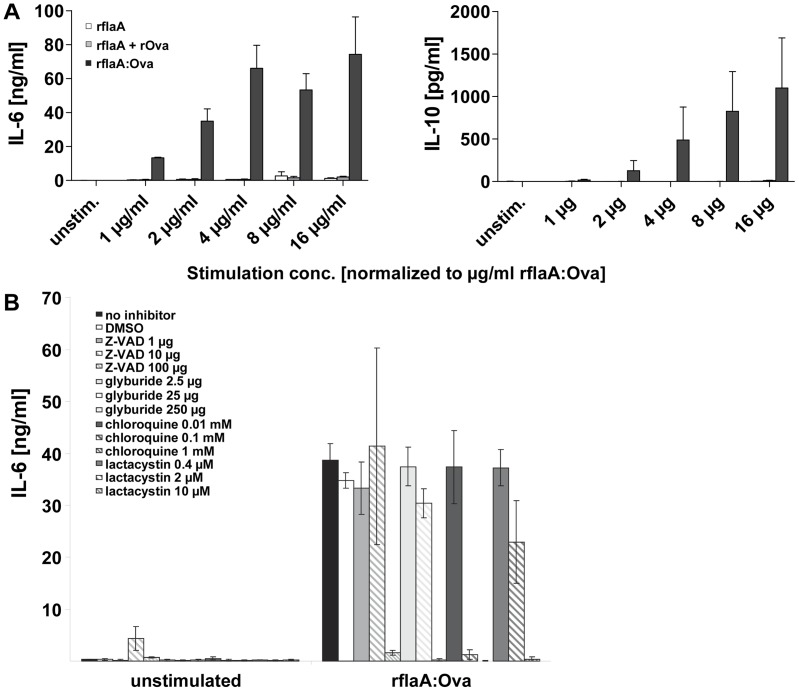
flaA:Ova mediated IL-6 secretion depends on both proteasome and inflammasome activation. BALB/c mDC were stimulated with the indicated equimolar amounts of rflaA, rflaA+rOva and rflaA:Ova for 24 h and the resulting IL-6 and IL-10 secretion in the culture supernatants was analyzed by ELISA (**A**). BALB/c mDC were incubated with either the inflammasome inhibitors Z-VAD-FMK and glyburide (or the proteasome inhibitor lactacystin) for 2 h (**B**). Subsequently mDC were stimulated for 22 h with 4 µg of rflaA:Ova. Supernatants were analyzed for IL-6 secretion via ELISA. Results are mean values of three independent experiments ± SD.

Moreover, in line with the stronger uptake of the fusion proteins into mDC (Fig. 3B), stimulation with equimolar amounts of the fusion protein resulted in a strongly increased cytokine secretion (108-fold higher IL-6, and 488-fold higher IL-10 secretion for the 4 µg/ml rflaA:Ova concentration) compared to rflaA alone or the mixture of both components (Fig. 5A). Here, application of chloroquine, an inhibitor of lysosomal acidification [25], which impedes the fusion of endosomes and lysosomes and subsequent lysosomal protein degradation [26] dose-dependently inhibited rflaA:Ova mediated mDC activation, depicted by rflaA:Ova induced IL-6 (Fig. 5B) and IL-10 secretion [27].

To study the involvement of either inflammasome or proteasome in mDC activation by rflaA:Ova, mDC were preincubated with respective inhibitors before stimulation with rflaA:Ova. Here, the rflaA:Ova induced IL-6 secretion by mDC was suppressed in a dose-dependent manner by the application of both inflammasome (Z-VAD-FMK, glyburide) and proteasome (lactacystin) inhibitors (Fig. 5B). In this experiment, no dose-dependent effect of the used inhibitors on rflaA:Ova-induced IL-10 secretion was observed.

### The Immune Modulating Properties of rflaA:Ova are Mediated via a TLR5-dependent IL-10 Secretion

**Figure 6:**
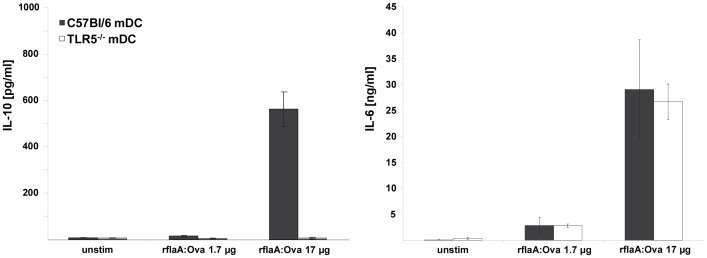
flaA:Ova induced IL-10 secretion from mDC is abrogated in TLR5 deficient mice. C57BL/6 and TLR5^−/−^ mDC were stimulated with rflaA:Ova. Levels of IL-6 and IL-10 were analyzed by ELISA after 72 h. Results are means ± SD of two independent experiments.

**Figure 7:**
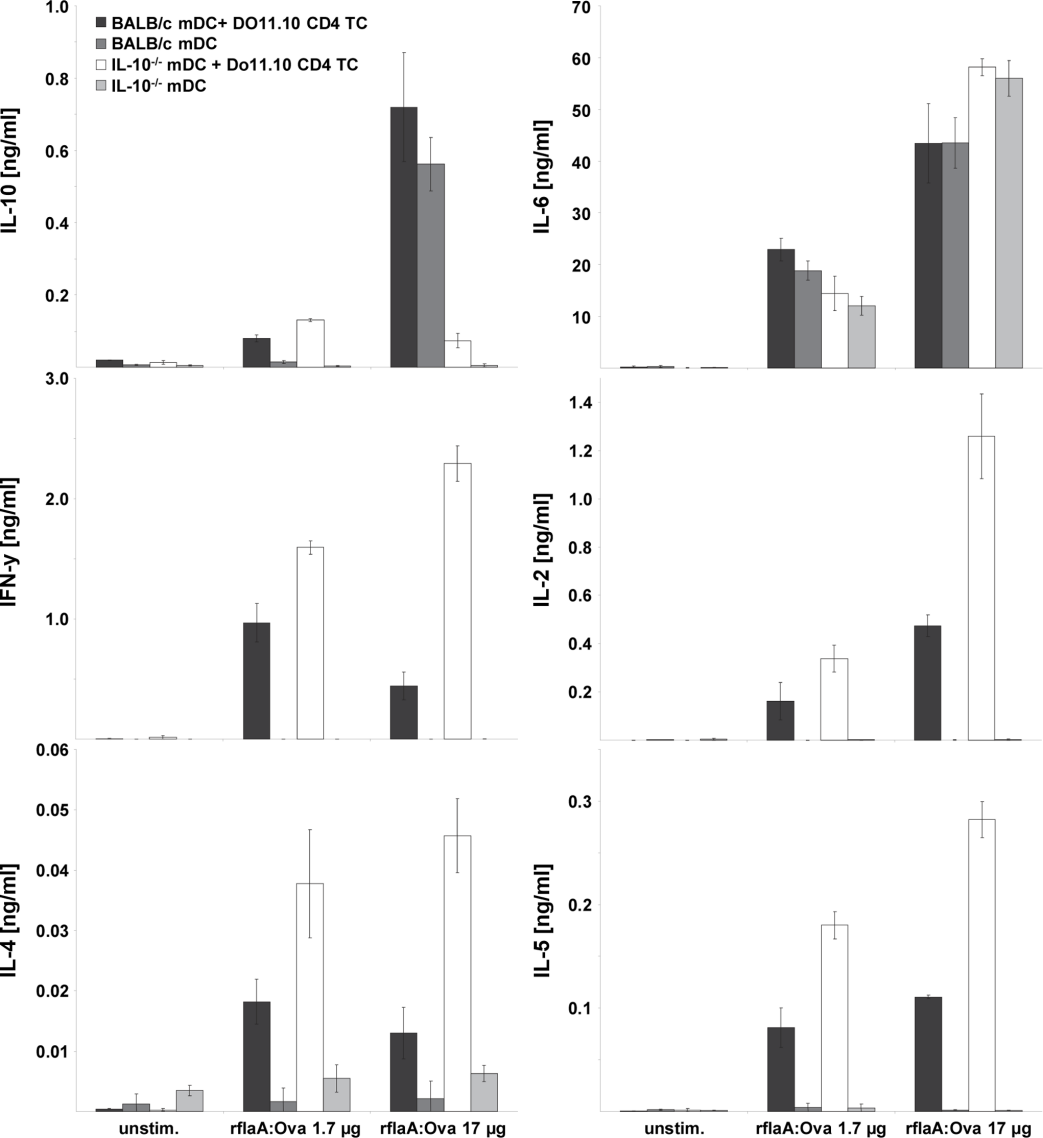
Suppression of TH1 and TH2 cytokine secretion by flaA:OVA is mediated by IL-10. BALB/c mDC derived from wt and IL-10^−/−^ mice were co-cultured with DO11.10 CD4^+^ T cells and stimulated with rflaA:Ova, respectively. Levels of IL-2 in the culture supernatants were analyzed after 24 h, levels of IL-4, IL-5, IL-6, IL-10 and IFN-y were analyzed after 72 h. Results are means ± SD of two independent experiments.

Cytokine secretion upon activation of mDC by rflaA:Ova was analyzed in order to investigate whether the enhanced internalization and the enforced degradation of the Ova fusion part [9], [19] were associated with the observed immune modulation *in vivo* (Fig. 6 and 7). Upon stimulation with the fusion protein, substantial levels of immune suppressive IL-10 and proinflammatory IL-6 were detectable in supernatants of bone marrow derived mDC from wild type (wt) mice (Fig. 6). When using TLR5^−/−^ mDC, the rflaA:Ova induced IL-10 secretion was strongly reduced, whereas IL-6 secretion remained unaffected (Fig. 6), demonstrating the IL-10 secretion to be TLR5 mediated and IL-6 secretion to be TLR5 independent. In this context, a potential contamination of the fusion protein with other non-protein stimulants was excluded by proteinase K digestion which resulted in strongly reduced IL-6 and IL-10 secretion compared to the undigested control (data not shown).

To further evaluate the impact of rflaA:Ova induced, TLR5-dependent IL-10 secretion on Ova-specific T cell responses, co-culture experiments were performed using BALB/c wt mDC or IL-10^−/−^ mDC and Ova-specific DO11.10 CD4^+^ T cells (Fig. 7). In line with previous results stimulation of wt mDC with rflaA:Ova resulted in predominant IL-10 secretion, whereas no IL-10 was detected in IL-10^−/−^ mDC controls. These results confirm, that most of the IL-10 secretion induced upon rflaA:Ova stimulation was attributed to the mDC population and not to the T cells [14]. In contrast to the experimental setting using wt mDCs, the lack of rflaA:Ova mediated IL-10 secretion resulted in strongly enhanced levels of both TH1 (IL-2∶2.65 fold higher and IFN-y: 5.2 fold higher) and TH2 (IL-4∶3.46 fold higher, IL-5∶2.56 fold higher) cytokine production upon rflaA:Ova (17 µg/ml) stimulation in IL-10^−/−^ mDC:DO11.10 T cell co-cultures (Fig. 7). In contrast, the rflaA:Ova induced IL-6 secretion by mDC was unaffected by IL-10 deficiency of the APC (Fig. 7).

### rflaA:Ova-induced IL-6 and IL-10 secretion from mDC are Mediated by Different Signaling Pathways

**Figure 8:**
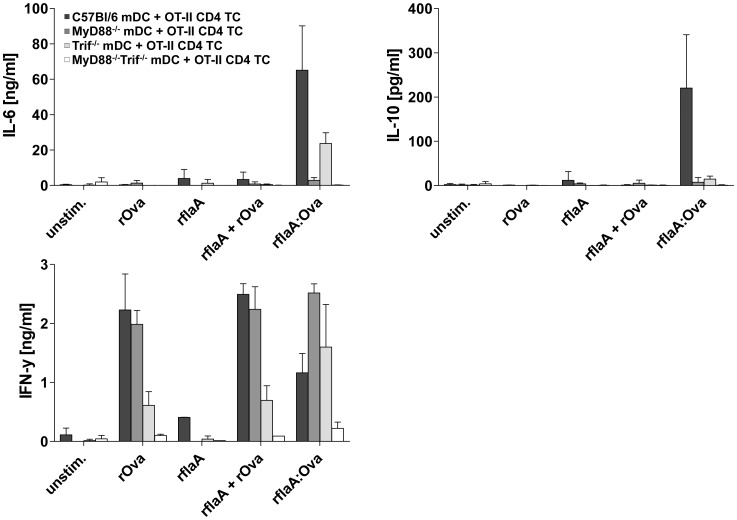
rflaA:Ova-induced IL-6 and IL-10 secretion from mDC are mediated by different signaling pathways. C57BL/6 mDC derived from C57BL/6 wt, MyD88^−/−^, Trif^−/−^, or MyD88^−/−^Trif^−/−^ mice were co-cultured with OT-II CD4^+^ T cells and stimulated with equimolar amounts of rOva, rflaA, rflaA+rOva, or rflaA:Ova, respectively. Levels of IL-6, IL-10, and IFN-y in the culture supernatants were analyzed after 72 h. Results are means ± SD of two independent experiments. Please note that results from C57BL/6 and Myd88^−/−^ cells were already published in Schülke *et al.*, 2011 JACI.

To further investigate the mechanism of rflaA:Ova induced cytokine secretion we differentiated mDC from either MyD88-, Trif- or MyD88/Trif-deficient mice and compared the rflaA:Ova-induced cytokine secretion to mDC derived from C57Bl/6 wild type control animals (Fig. 8). Here, we observed the rflaA:Ova induced IL-10 secretion to be abrogated in both MyD88- and Trif-deficient cells, whereas residual IL-6 secretion was still detectable in Trif-deficient but not in MyD88-deficient mDC (Fig. 8). In co-culture with Ova-specific OT-II CD4 T cells, only deletion of MyD88 was sufficient to reverse the rflaA:Ova induced suppression of TH1 cytokine secretion (Fig. 8). Here, deficiency of the mDC for Trif showed reduced levels of IFN-y in response to rOva and rflaA plus rOva stimulation (Fig. 8), whereas co-cultures incorporating MyD88^−/−^Trif^−/−^ mDC did not produce IFN-y (Fig. 8).

### rflaA:Ova Induces Stronger Activation of Human Monocytes than the Mixture of both Proteins

**Figure 9:**
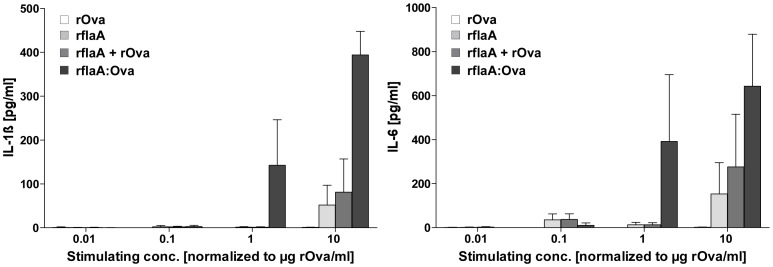
rflaA:Ova induces strong cytokine secretion from human monocytes. Monocyte activation test was performed using 5 µl of fresh blood stimulated with the indicated, equimolar protein amounts for 24 h. Supernatants were analysed for IL-1ß and IL-6 cytokine secretion by ELISA. Results are mean values ± SD for four different donors.

To test whether rflaA:Ova even mediates activation of human antigen presenting cells monocyte activation tests were performed using peripheral blood from healthy human donors (Fig. 9). Here, in line with the results presented in the murine system, stimulation of human monocytes with the fusion protein resulted in a dose-dependent, and stronger IL-1ß and IL-6 secretion than observed with either flaA alone or both proteins applied as a mixture (Fig. 9).

## Discussion

In an experimental mouse model of Ova-induced intestinal allergy [20] both i.p and i.n. vaccination with the fusion protein were shown to efficiently prevent allergic sensitization. Vaccination resulted in a reduction of clinical symptoms, reduced Ova-specific IgE levels and a predominant induction of Ova-specific IgG2a antibodies. Here, in line with flagellin acting as a mucosal adjuvant [11], [14], [28], an intranasal application of the fusion protein was shown to be even more efficient in suppressing allergy eliciting IgE antibodies. Therefore, flagellin containing fusion proteins hold great potential as mucosal vaccines for the treatment of allergic diseases.

To further elucidate the mechanisms by which flagellin fusion proteins induce such strong protective immune responses, we characterized the effects of rflaA:Ova stimulation on murine mDCs as target cells for a successful vaccination [29]. Light scattering analysis revealed that both flaA and rflaA:Ova form high molecular aggregates in solution. This aggregation is probably caused by spontaneous self-assembly of flagellin, which is known from the biosynthesis of flagella. Whereas for rflaA these aggregates are non-covalent in nature, for rflaA:Ova aggregation further strengthened by intermolecular cysteine bonds (between the cysteine residues of the Ova fusion part), was observed under non reducing conditions (unpublished observation). Moreover, uptake experiments revealed the fusion protein to be taken up approx. 10 times more efficiently than the equimolar mixture of both components. Using mDC derived from TLR5-deficient animals the uptake of flaA and rflaA:Ova but not rOva was shown to be in part mediated by TLR5. Since protein uptake was determined 15 minutes post stimulation we think that the observed stronger uptake of the fusion protein is mediated by receptor mediated uptake and at this time point not yet the result of the induced TLR5-signalling or stimulation-induced TLR5 upregulation as described in Schülke *et al.,* 2011 [9]. Based on these results we conclude, that the enhanced uptake of the fusion protein depends on both TLR5-facilitated receptor mediated uptake as well as an unspecific uptake of aggregates. In line with these results a flagellinC:EGFP fusion protein was found to be efficiently internalized, processed, and presented by mouse APC [30]. Consequently, BALB/c mice immunized with the flagellinC:EGFP construct developed specific CD4 and CD8 T cell responses against the otherwise non-immunogenic EGFP [30]. We showed that covalent rflaA:Ova aggregation may likely influence antigen uptake, as well as subsequent processing and presentation by DC and therefore the immunogenicity of the fusion construct. In line with these results stimulation with equimolar amounts of the fusion protein in direct comparison to rflaA alone or the mixture of both proteins was shown to result in a strongly enhanced secretion of IL-6 and IL-10 from mDC. These results are in accordance with both our own previous results [9], [27] and the growing evidence that flagellin containing fusion proteins have superior immune modulating capacities over the simple mixture of both components shown by others [9]–[14]. In line with this, it has been reported that the mixture of TLR-ligands and Ova (in comparison to covalent fusion of both) has negligible impact on the processing of Ova (Khan *et al.*, 2007 JBC).

The rflaA:Ova-induced IL-6 secretion from TLR5^−/−^ which was unchanged compared to wt mDC as well as the still observed stronger uptake of rflaA:Ova in TLR5-deficient mDC suggest that internalisation of the fusion likely is mediated by both TLR5-dependent and TLR5-independent mechanisms. In line with this, Cuadros *et al.* speculated that the flagellin:EGFP fusion protein does not only activate APC via TLR5 but also by increased internalization of the complex and subsequent differences in processing and presentation [30]. Consistently, it was hypothesized that the size of (fusion) proteins influences processing by the APC, with high molecular weight proteins being slightly more effectively processed [30]. Concordantly, Zaborsky and coworkers showed that Bet v 1 dimerization induced enhanced antigen uptake and DC activation [31]. Moreover, Legge *et al.* reported that strong aggregation observed for an Ig chimera carrying the encephalitogenic proteolipid protein (PLP) 1 induced IL-10 production by macrophages and DC, whereas the non-aggregated form failed to induce IL-10 secretion [32]. Since aggregate sizes were comparable for rflaA and rflaA:Ova but uptake levels were clearly different we hypothesize, that other mechanisms besides the observed aggregation contribute to the fusion proteins immune modulating capacity. Here, it is noteworthy that the fusion proteins uptake may be strongly facilitated by its binding to TLR5, which was recently shown to promote flagellin uptake as endocytotic receptor [33], whereas for non-glycosylated rOva no corresponding receptor is described so far. In line with this, in a previous study we found that mDC stimulation with rflaA:Ova resulted in a strong upregulation of TLR5 whereas stimulation with rflaA plus rOva in equimolar concentrations did not [9]. This difference in the inducible TLR5 expression might also account for the observed differences in protein uptake, despite the comparable size of the protein aggregates, between rflaA and rflaA:Ova. In line with the increased uptake of the fusion protein, pretreatment with chloroquine which blocks lysosomal acidification and therefore subsequent lysosomal protein degradation [25], [26] was able to prevent fusion protein mediated cell activation. Additionally, both inhibition of inflammasome and proteasome activation were sufficient to abrogate rflaA:Ova mediated IL-6 secretion from mDC, suggesting the involvement of both processes in fusion protein mediated cell activation.

Taken together these results suggest, that stimulation with rflaA:Ova results in a stronger uptake of the fusion protein, possibly mediated by a yet unknown receptor independent mechanism (e.g. phagocytosis) and which is amplified by additional TLR5-mediated endocytosis. Additionally, uptake of Ova antigen conjugated to flagellin resulted in different processing of the conjugated Ova as shown by microsomal digestion performed with microsomes isolated from unstimulated mDC. Here, a faster degradation of rflaA:Ova in comparison to rOva alone was observed, possibly due to enforced internalization and different accessibility to protease cleavage sites. Taken together, these processes may explain both the faster generation of Ova-derived peptides and the broader spectrum of Ova-derived peptides generated. In this experimental setting structural changes of Ova, induced upon fusion to another protein, likely modulates its processing by changing the accessibility of peptide bonds to microsomal cathepsins. However, the specific peptides presented by MHC class II and their role for the activation of Ova-specific T cells in the context of rflaA:Ova mediated mDC activation remain a matter of further studies.

In our previous study we observed suppression of both TH1 and TH2 cytokine production from Ova-specific CD4 T cells upon co-culture with rflaA:Ova stimulated mDC possibly attributed to the strong IL-10 secretion from mDC [9]. Here, rflaA:Ova induced IL-10 secretion was shown to be TLR5-dependent since TLR5^−/−^ mDC failed to produce IL-10 upon rflaA:Ova stimulation. In contrast to this, the rflaA:Ova-induced secretion of IL-6 was found to be TLR5-independent, suggesting that fusion protein induced IL-6 and IL-10 secretion are regulated by different signaling pathways. This assumption was strengthened using mDC from MyD88-, Trif- and MyD88/Trif-deficient mice. We observed the rflaA:Ova induced IL-10 secretion to be abrogated in both MyD88- and Trif-deficient cells, whereas residual IL-6 secretion was still detectable in Trif deficient but not in MyD88 deficient mDC. In co-culture with Ova-specific OT-II CD4 T cells, only deletion of MyD88 was sufficient to reverse the rflaA:Ova-induced suppression of TH1 cytokine secretion [19]. Co-cultures incorporating MyD88/Trif double deficient mDC did not produce IFN-y, probably due to the complete lack of mDC derived costimulatory signals. This difference in rflaA:Ova-induced IL-6 and IL-10 signalling is supported by our finding that only rflaA:Ova-induced IL-6 (but not IL-10) secretion was dose-dependently inhibited by inhibitors of either inflammasome or proteasome.

To further elucidate the contribution of the enhanced IL-10 production to the immune modulation observed in our model we used mDC derived from IL-10 deficient mice in co-culture experiments with Ova-specific DO11.10 CD4^+^ T cells. Here we could show that the rflaA:Ova induced suppression of TH1 and TH2 cytokine production was reversed when using IL-10 deficient mDC. These results clearly demonstrate the observed immune modulation to be mediated by the mDC derived TLR5-dependent IL-10 secretion upon rflaA:Ova mediated mDC activation.

Taken together the data presented in this study are able to further explain the increased immunogenicity observed for this and other flagellin:antigen fusion proteins *in vivo* [9]–[14]. We observed a much stronger activation of professional antigen presenting cells when stimulated with the fusion protein compared to rflaA alone or the mixture of both proteins [9], [27]. Here, *in vitro* the rflaA:Ova-induced IL-10 secretion was the dominant cytokine leading to a suppression of TH1 and TH2 responses. In conclusion with this the rflaA:Ova-induced anti-inflammatory IL-10 secretion seems to be mediated at least in part by a mechanism different than the one observed for the inflammasome- and proteasome-dependent, secretion of proinflammatory IL-6. Moreover, *in vivo* we observed a prominent suppression of allergic sensitzation and allergic symptoms with significantly reduced Ova-specific IgE titers paralleled by a significant induction of potentially blocking Ova-specific IgG2a antibodies. Therefore, we hypothesize that vaccination with the fusion protein results in a strong, initial activation of the innate immune response mediated by the observed stronger uptake and the TLR5-induced cell activation. Here, a covalent fusion of flagellin and antigen has clearly superior immune activating properties compared to the simple mixture of both components. The strong cell activation is subsequently accompanied by a TLR5- and IL-10 mediated counter regulation resulting in clinical tolerance towards the fused antigen (by the suppression of TH1 and TH2 responses *in vitro* and the observed suppression of CD4 and CD8 T cell activation *in vivo* [9]). The fact that we observed a strong induction of Ova-specific IgG2a antibodies upon vaccination with the fusion protein suggests, that there might be other cell types and mechanisms involved in the immune responses triggered by flagellin fusion proteins. In this context further studies aim at identifying and characterizing the contribution of other TLR5-positive cell types to the observed effects (e.g. macrophages or epithelial cells).

Finally, using a human monocyte activation test we observed that stimulation of monocytes with the fusion protein resulted in both stronger IL-1ß and IL-6 secretion than observed with either flaA alone or both proteins applied as a mixture. These results provide first evidence for the transferability of the results derived from mouse experiments to the human system. Here, the enhanced cytokine secretion from human monocytes is likely of antigen unspecific nature, but obviously mediated by the structural features of the fusion protein and its (flagellin-mediated) immune stimulating abilities. Further studies will address allergen-specific immune responses in allergic compared to non-allergic donors.

In summary, our study further elucidated the mechanisms and the potential of TLR5 ligand flagellin:allergen fusion proteins in the context of allergies. We could show that aggregation of the fusion protein is associated with a stronger uptake and more efficient microsomal digestion than observed for rOva alone. Moreover, stimulation with rflaA:Ova resulted in a strong inflammasome and proteasome dependent mDC activation. In contrast to IL-6 rflaA:Ova induced IL-10 secretion from mDC was shown to be TLR5-dependent and to be sufficient for the suppression of both TH1 and TH2 cytokine secretion observed in Ova-specific CD4+ T cells *in vitro*. In a murine model of Ova-induced intestinal allergy intranasal application of the fusion protein was shown to protect even more from allergic sensitization than the already established i.p. route. In conclusion, conjugated proteins consisting of flaA and an allergen are promising vaccine candidates for mucosal administration in specific immunotherapy of allergies.

## Supporting Information

Figure S1 **Mouse phenotype and appearance of faeces used to calculate symptom sores.**
(TIF)

Figure S2 **Prophylactic vaccination with rflaA:Ova induces IgG2a and suppresses IgE production.** Sera of control (PBS→PBS→NF) and Ova-sensitized and EW-challenged animals (Ova/A→EW) were collected after immunization with Ova on day 7 (**A+C**) or after the second immunization with Ova on day 21 (**B+D**) and analyzed for Ova-specific IgE **(A+B)** and Ova-specific IgG2a (**C+D**) antibody levels by ELISA (n = 6 mice per group).
(TIF)

## References

[1] Hayashi F, Smith KD, Ozinsky A, Hawn TR, Yi EC, et al (2001) The innate immune response to bacterial flagellin is mediated by Toll-like receptor 5. Nature 410: 1099–1103. 11323673 10.1038/35074106

[2] Gewirtz AT, Navas TA, Lyons S, Godowski PJ, Madara JL (2001) Cutting edge: bacterial flagellin activates basolaterally expressed TLR5 to induce epithelial proinflammatory gene expression. J Immunol 167: 1882–1885. 11489966 10.4049/jimmunol.167.4.1882

[3] Sierro F, Dubois B, Coste A, Kaiserlian D, Kraehenbuhl JP, et al (2001) Flagellin stimulation of intestinal epithelial cells triggers CCL20-mediated migration of dendritic cells. Proc Natl Acad Sci U S A 98: 13722–13727. 11717433 10.1073/pnas.241308598 PMC61108

[4] Wilson RH, Maruoka S, Whitehead GS, Foley JF, Flake GP, et al (2012) The Toll-like receptor 5 ligand flagellin promotes asthma by priming allergic responses to indoor allergens. Nat Med 18: 1705–1710 nm.2920 [pii];10.1038/nm.2920 [doi]. 23064463 10.1038/nm.2920 PMC3493750

[5] Didierlaurent A, Ferrero I, Otten LA, Dubois B, Reinhardt M, et al (2004) Flagellin promotes myeloid differentiation factor 88-dependent development of Th2-type response. J Immunol 172: 6922–6930. 15153511 10.4049/jimmunol.172.11.6922

[6] Way SS, Thompson LJ, Lopes JE, Hajjar AM, Kollmann TR, et al (2004) Characterization of flagellin expression and its role in Listeria monocytogenes infection and immunity. Cell Microbiol 6: 235–242. 14764107 10.1046/j.1462-5822.2004.00360.x

[7] Lee SE, Koh YI, Kim MK, Kim YR, Kim SY, et al (2008) Inhibition of airway allergic disease by co-administration of flagellin with allergen. J Clin Immunol 28: 157–165. 18026856 10.1007/s10875-007-9138-3

[8] Wilson RH, Maruoka S, Whitehead GS, Foley JF, Flake GP, et al (2012) The Toll-like receptor 5 ligand flagellin promotes asthma by priming allergic responses to indoor allergens. Nat Med 18: 1705–1710 nm.2920 [pii];10.1038/nm.2920 [doi]. 23064463 10.1038/nm.2920 PMC3493750

[9] Schuelke S, Burggraf M, Waibler Z, Wangorsch A, Wolfheimer S, et al (2011) A fusion protein of flagellin and ovalbumin suppresses the TH2 response and prevents murine intestinal allergy. J Allergy Clin Immunol 128: 1340–1348 S0091–6749(11)01157–2 [pii];10.1016/j.jaci.2011.07.036 [doi]. 21872305 10.1016/j.jaci.2011.07.036

[10] Bates JT, Uematsu S, Akira S, Mizel SB (2009) Direct stimulation of tlr5+/+ CD11c+ cells is necessary for the adjuvant activity of flagellin. J Immunol 182: 7539–7547. 19494277 10.4049/jimmunol.0804225 PMC3770462

[11] Lee SE, Kim SY, Jeong BC, Kim YR, Bae SJ, et al (2006) A bacterial flagellin, Vibrio vulnificus FlaB, has a strong mucosal adjuvant activity to induce protective immunity. Infect Immun 74: 694–702 74/1/694 [pii];10.1128/IAI.74.1.694–702.2006 [doi]. 16369026 10.1128/IAI.74.1.694-702.2006 PMC1346682

[12] McDonald WF, Huleatt JW, Foellmer HG, Hewitt D, Tang J, et al (2007) A West Nile virus recombinant protein vaccine that coactivates innate and adaptive immunity. J Infect Dis 195: 1607–1617. 17471430 10.1086/517613

[13] Mizel SB, Graff AH, Sriranganathan N, Ervin S, Lees CJ, et al (2009) Flagellin-F1-V fusion protein is an effective plague vaccine in mice and two species of nonhuman primates. Clin Vaccine Immunol 16: 21–28. 18987167 10.1128/CVI.00333-08 PMC2620661

[14] Mizel SB, Bates JT (2010) Flagellin as an adjuvant: cellular mechanisms and potential. J Immunol 185: 5677–5682. 21048152 10.4049/jimmunol.1002156 PMC3756556

[15] Weimer ET, Lu H, Kock ND, Wozniak DJ, Mizel SB (2009) A fusion protein vaccine containing OprF epitope 8, OprI, and type A and B flagellins promotes enhanced clearance of nonmucoid Pseudomonas aeruginosa. Infect Immun 77: 2356–2366. 19349426 10.1128/IAI.00054-09 PMC2687341

[16] Song L, Nakaar V, Kavita U, Price A, Huleatt J, et al (2008) Efficacious recombinant influenza vaccines produced by high yield bacterial expression: a solution to global pandemic and seasonal needs. PLoS ONE 3: e2257. 18493310 10.1371/journal.pone.0002257 PMC2373928

[17] Weimer ET, Ervin SE, Wozniak DJ, Mizel SB (2009) Immunization of young African green monkeys with OprF epitope 8-OprI-type A- and B-flagellin fusion proteins promotes the production of protective antibodies against nonmucoid Pseudomonas aeruginosa. Vaccine 27: 6762–6769. 19744586 10.1016/j.vaccine.2009.08.080

[18] Huleatt JW, Jacobs AR, Tang J, Desai P, Kopp EB, et al (2007) Vaccination with recombinant fusion proteins incorporating Toll-like receptor ligands induces rapid cellular and humoral immunity. Vaccine 25: 763–775. 16968658 10.1016/j.vaccine.2006.08.013

[19] Schuelke S, Waibler Z, Mende MS, Zoccatelli G, Vieths S, et al (2010) Fusion protein of TLR5-ligand and allergen potentiates activation and IL-10 secretion in murine myeloid DC. Mol Immunol 48: 341–350 S0161–5890(10)00507–9 [pii];10.1016/j.molimm.2010.07.006 [doi]. 20965571 10.1016/j.molimm.2010.07.006

[20] Burggraf M, Nakajima-Adachi H, Hachimura S, Ilchmann A, Pemberton AD, et al (2011) Oral tolerance induction does not resolve gastrointestinal inflammation in a mouse model of food allergy. Mol Nutr Food Res 55: 1475–1483 10.1002/mnfr.201000634 [doi]. 21714123 10.1002/mnfr.201000634

[21] Egger M, Jurets A, Wallner M, Briza P, Ruzek S, et al (2011) Assessing protein immunogenicity with a dendritic cell line-derived endolysosomal degradome. PLoS ONE 6: e17278 10.1371/journal.pone.0017278 [doi]. 21359181 10.1371/journal.pone.0017278 PMC3040223

[22] Delamarre L, Pack M, Chang H, Mellman I, Trombetta ES (2005) Differential lysosomal proteolysis in antigen-presenting cells determines antigen fate. Science 307: 1630–1634 307/5715/1630 [pii];10.1126/science.1108003 [doi]. 15761154 10.1126/science.1108003

[23] Hoffmann S, Peterbauer A, Schindler S, Fennrich S, Poole S, et al (2005) International validation of novel pyrogen tests based on human monocytoid cells. J Immunol Methods 298: 161–173 S0022–1759(05)00024–4 [pii];10.1016/j.jim.2005.01.010 [doi]. 15847806 10.1016/j.jim.2005.01.010

[24] Montag T, Spreitzer I, Loschner B, Unkelbach U, Flory E, et al (2007) Safety testing of cell-based medicinal products: opportunities for the monocyte activation test for pyrogens. ALTEX 24: 81–89. 10.14573/altex.2007.2.81 17728974

[25] Steinman RM, Mellman IS, Muller WA, Cohn ZA (1983) Endocytosis and the recycling of plasma membrane. J Cell Biol 96: 1–27. 6298247 10.1083/jcb.96.1.1 PMC2112240

[26] Shintani T, Klionsky DJ (2004) Autophagy in health and disease: a double-edged sword. Science 306: 990–995 306/5698/990 [pii];10.1126/science.1099993 [doi]. 15528435 10.1126/science.1099993 PMC1705980

[27] Schuelke S, Waibler Z, Mende MS, Zoccatelli G, Vieths S, et al (2010) Fusion protein of TLR5-ligand and allergen potentiates activation and IL-10 secretion in murine myeloid DC. Mol Immunol 48: 341–350 S0161–5890(10)00507–9 [pii];10.1016/j.molimm.2010.07.006 [doi]. 20965571 10.1016/j.molimm.2010.07.006

[28] Skountzou I, Martin MP, Wang B, Ye L, Koutsonanos D, et al (2010) Salmonella flagellins are potent adjuvants for intranasally administered whole inactivated influenza vaccine. Vaccine 28: 4103–4112. 19654062 10.1016/j.vaccine.2009.07.058 PMC3187848

[29] Uematsu S, Akira S (2009) Immune responses of TLR5(+) lamina propria dendritic cells in enterobacterial infection. J Gastroenterol. 10.1007/s00535-009-0094-y 19547909

[30] Cuadros C, Lopez-Hernandez FJ, Dominguez AL, McClelland M, Lustgarten J (2004) Flagellin fusion proteins as adjuvants or vaccines induce specific immune responses. Infect Immun 72: 2810–2816. 15102791 10.1128/IAI.72.5.2810-2816.2004 PMC387897

[31] Zaborsky N, Brunner M, Wallner M, Himly M, Karl T, et al (2010) Antigen aggregation decides the fate of the allergic immune response. J Immunol 184: 725–735. 19995902 10.4049/jimmunol.0902080 PMC2968749

[32] Legge KL, Min B, Bell JJ, Caprio JC, Li L, et al (2000) Coupling of peripheral tolerance to endogenous interleukin 10 promotes effective modulation of myelin-activated T cells and ameliorates experimental allergic encephalomyelitis. J Exp Med 191: 2039–2052. 10859329 10.1084/jem.191.12.2039 PMC2193208

[33] Letran SE, Lee SJ, Atif SM, Uematsu S, Akira S, et al (2011) TLR5 functions as an endocytic receptor to enhance flagellin-specific adaptive immunity. Eur J Immunol 41: 29–38 10.1002/eji.201040717 [doi]. 21182074 10.1002/eji.201040717 PMC3652676

