# Supplementary figures and images for: Prevention of Intestinal Allergy in Mice by rflaA:Ova Is Associated with Enforced Antigen Processing and TLR5-Dependent IL-10 Secretion by mDC
Source: PLoS One. 2014 Feb 7;9(2):e87822. doi: 10.1371/journal.pone.0087822 (PMC3917841; doi:10.1371/journal.pone.0087822)

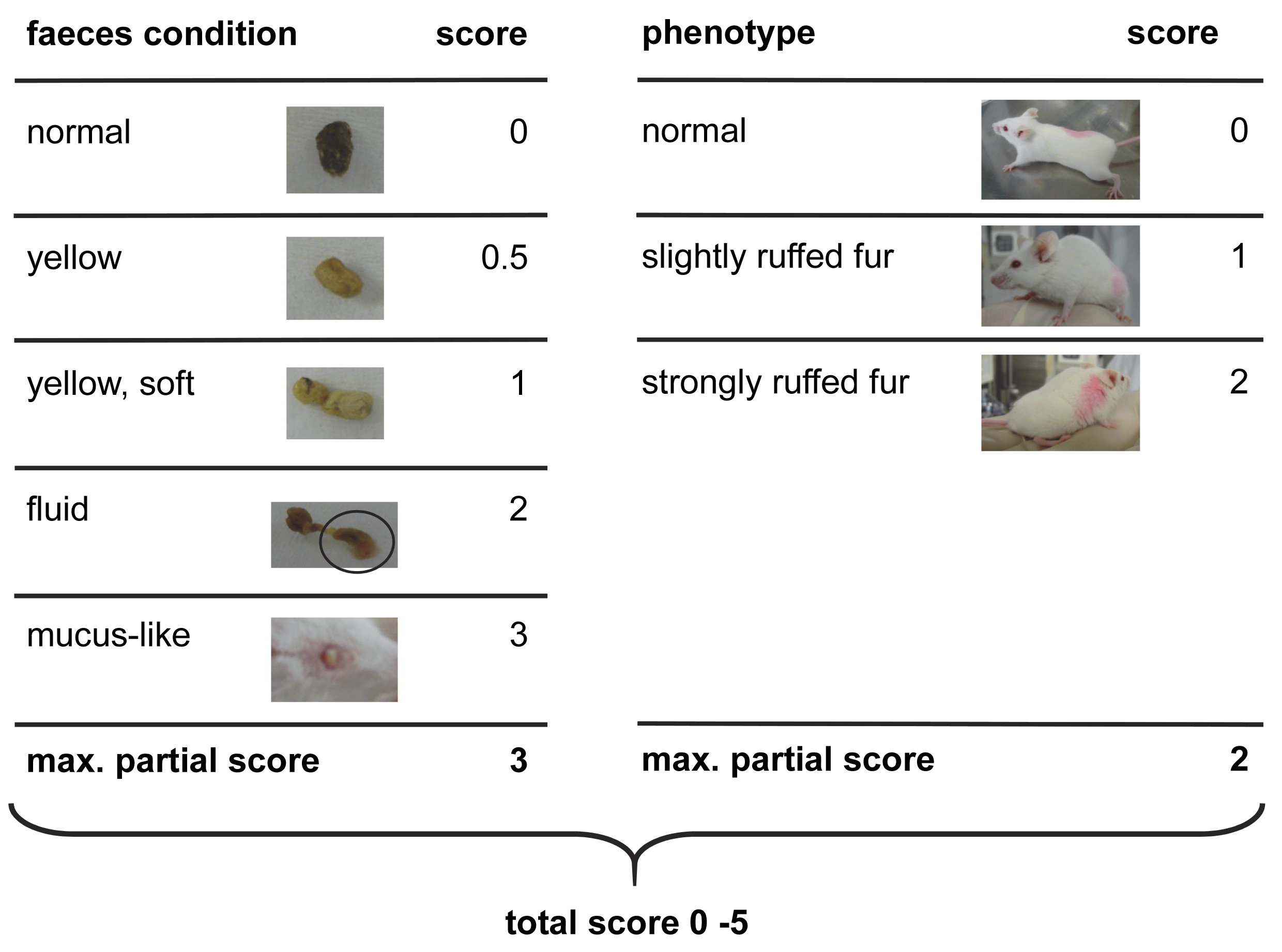

Supplement: Figure S1 — Mouse phenotype and appearance of faeces used to calculate symptom sores. (TIF) [file pone.0087822.s001.tif]

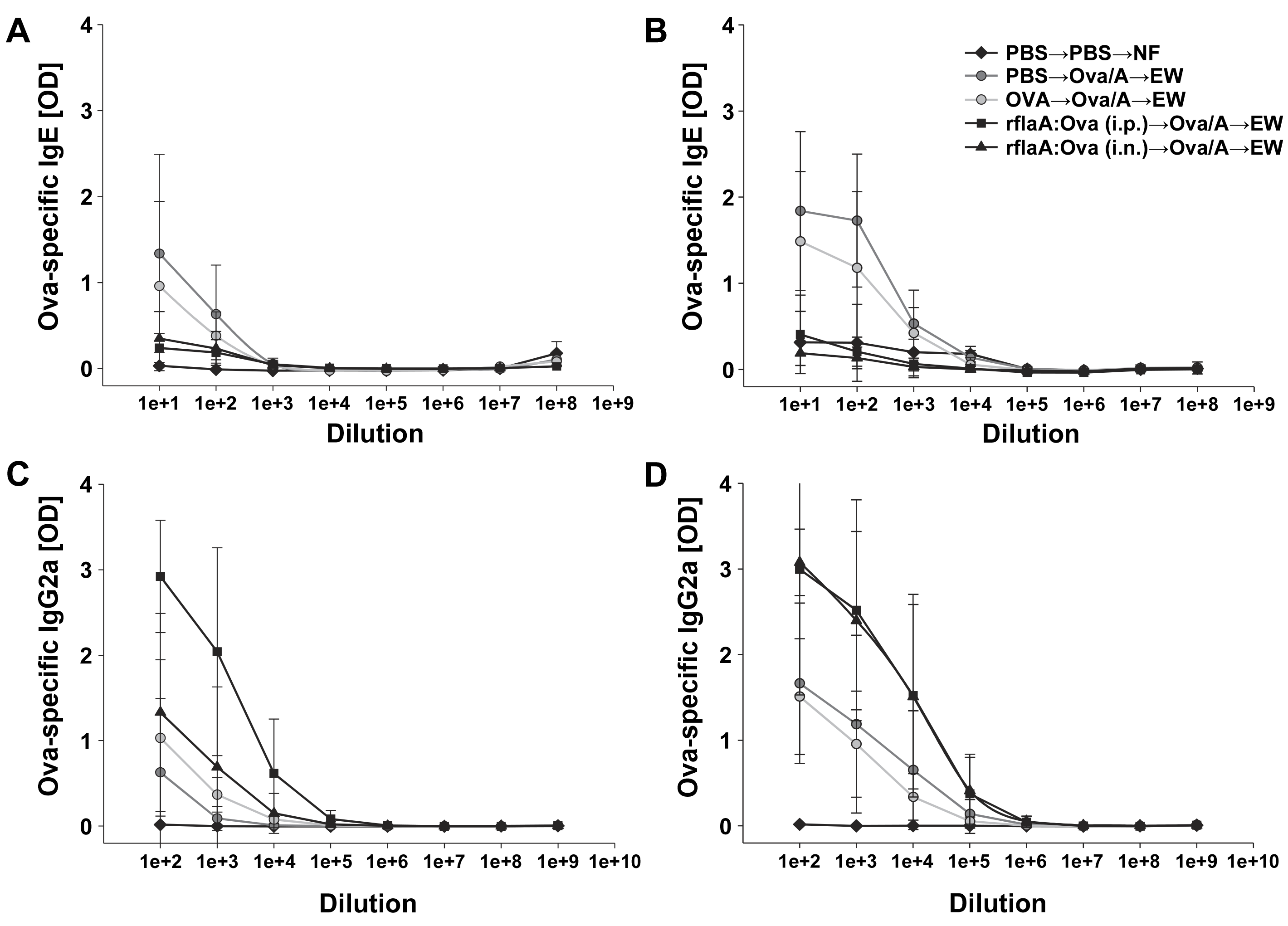

Supplement: Figure S2 — Prophylactic vaccination with rflaA:Ova induces IgG2a and suppresses IgE production. Sera of control (PBS→PBS→NF) and Ova-sensitized and EW-challenged animals (Ova/A→EW) were collected after immunization with Ova on day 7 (A+C) or after the second immunization with Ova on day 21 (B+D) and analyzed for Ova-specific IgE (A+B) and Ova-specific IgG2a (C+D) antibody levels by ELISA (n = 6 mice per group). (TIF) [file pone.0087822.s002.tif]
